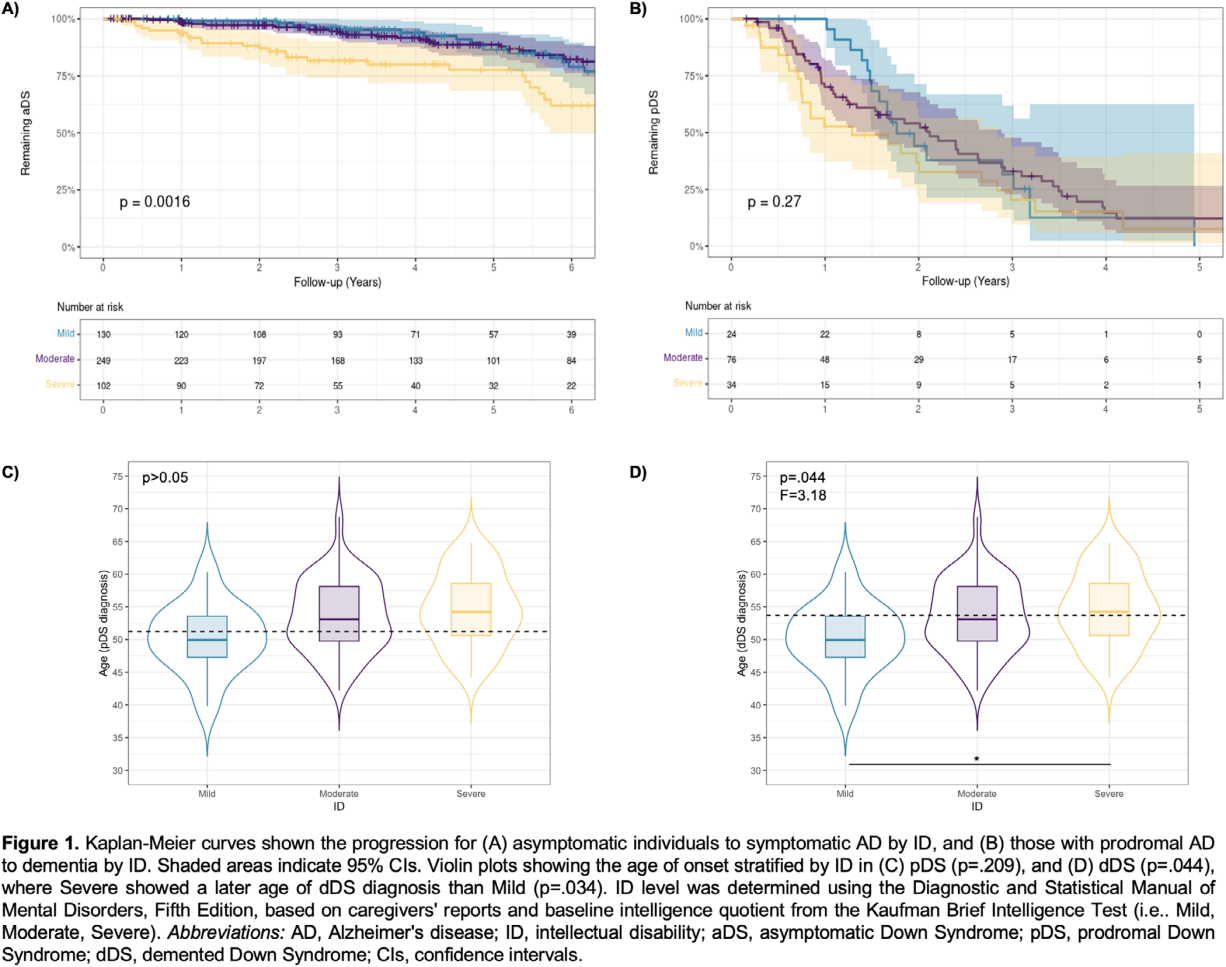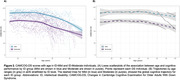# Alzheimer disease clinical progression by intellectual disability levels in Down syndrome

**DOI:** 10.1002/alz.086289

**Published:** 2025-01-09

**Authors:** Lídia Vaqué‐Alcázar, Laura Videla, María Carmona, Bessy Benejam, Isabel Barroeta, Laura Del Hoyo, Aida Sanjuan Hernandez, Íñigo Rodríguez‐Baz, José Enrique Arriola‐Infante, Javier Arranz, Lucía Maure‐Blesa, Alejandra O. Morcillo‐Nieto, Alexandre Bejanin, Alberto Lleo, David Bartrés‐Faz, Juan Fortea, Susana Fernandez

**Affiliations:** ^1^ Sant Pau Memory Unit, Hospital de la Santa Creu i Sant Pau, Biomedical Research Institute Sant Pau, Universitat Autònoma de Barcelona, Barcelona Spain; ^2^ Department of Medicine, Faculty of Medicine and Health Sciences, Institute of Neurosciences, University of Barcelona, Barcelona, Spain. Institut d’Investigacions Biomèdiques August Pi i Sunyer (IDIBAPS), Barcelona Spain; ^3^ Center for Biomedical Investigation Network for Neurodegenerative Diseases (CIBERNED), Madrid Spain; ^4^ Barcelona Down Medical Center, Fundació Catalana Síndrome de Down, Barcelona Spain; ^5^ Sant Pau Memory Unit, Hospital de la Santa Creu i Sant Pau, Biomedical Research Institute Sant Pau, Universitat Autònoma de Barcelona, Barcelona, Barcelona Spain; ^6^ Hospital de la Santa Creu i Sant Pau ‐ Biomedical Research Institute Sant Pau ‐ Autonomous University of Barcelona, Barcelona, Catalonia Spain; ^7^ Institut Guttmann, Institut Universitari de Neurorehabilitació adscrit a la Universitat Autònoma de Barcelona, Badalona, Barcelona Spain

## Abstract

**Background:**

The extended life expectancy in individuals with Down Syndrome (DS) has led to the emergence of age‐related diseases, with Alzheimer’s disease (AD) being particularly noteworthy due to its nearly full penetrance. The level of intellectual disability (ID), regarded as a proxy for cognitive reserve (CR), explains heterogeneity in cognitive and functional abilities. Despite this, there is a notable lack of exploration into the characterization of resilience factors and their potential influence on the progression along the AD continuum in this population. The main goal was to study whether premorbid ID levels (i.e., Mild, Moderate, Severe) could influence the AD clinical onset (prodromal [pDS] or dementia [dDS] diagnosis) and cognitive trajectories among asymptomatic DS (aDS).

**Method:**

We analyzed longitudinal data (> 6 months follow‐up) from a single‐center cohort study involving DS adults (aged >18 years) recruited at the Alzheimer‐Down Unit from the Catalan Down Syndrome Foundation and Hospital of Sant Pau. Clinical progression was evaluated using Kaplan‐Meier curves, ANOVA was conducted to test differences in the age of onset across ID groups, and cognitive trajectories (assessed with Cambridge Cognitive Examination for Older Adults with Down Syndrome [CAMCOG‐DS]) were analyzed using linear mixed‐effects models to explore age‐related effects by ID.

**Result:**

A total of 778 adults with DS (mean [SD] age, 43.32 [11.07] years; 361 women [46.4%]; aDS = 527, pDS = 86, dDS = 165) were included. Kaplan‐Meier curves showed that aDS with ID‐Severe progressed faster (p = .0016; Figure 1A), whereas no differences were found in the prodromal stage (p = 0.270; Figure 1B). While no significant differences were identified regarding the age of onset for pDS (Figure 1C), the ID‐Severe slightly showed a later age of dDS diagnosis than ID‐Mild (p = .044; Figure 1D). CAMCOG‐DS scores were higher for ID‐Mild than those with ID‐Moderate (mean [SE] difference, −19.24 [1.68]); p<.001). Nevertheless, there were no group differences among trajectories (Figure 2A), neither when estimated by different age ranges (Figure 2B).

**Conclusion:**

Despite the lack of evidence, there is an increasing interest in understanding how CR proxies contribute to cognitive resilience in DS. Further analyses including AD‐related biomarkers are essential to uncover the neurobiological mechanisms involved, which could significantly contribute to targeted primary prevention strategies.